# Estimating the role of single-nucleotide polymorphism (rs1800629)-308 G/A of TNF-alpha gene as genetic marker associated with angina pectoris in a sample of Iraqi patients

**DOI:** 10.1186/s43141-022-00454-w

**Published:** 2023-01-09

**Authors:** Shaimaa Y. Abdulfattah, Farah Thamer Samawi

**Affiliations:** 1 Molecular Genetics; Medical and Molecular Dept.; Biotechnology Research Center, Al-Nahrain University, Baghdad, Iraq; 2 Immunogenetics; Medical and Molecular Dept.; Biotechnology Research Center, Al-Nahrain University, Baghdad, Iraq

**Keywords:** Angina pectoris, Tumor necrosis factor, Genetic model, (rs1800629) -308G/A

## Abstract

**Background:**

Angina pectoris (AP) occurs when oxygen and other nutrients are insufficient to meet the metabolic needs of the heart muscle. Stable angina is the most common, while the unstable angina is less frequent. Tumor necrosis factor alpha (TNF-alpha) is a pleiotropic cytokine plays a vital function in the immune response regulation. TNF gene cluster contains many polymorphisms; the most commonly investigated polymorphism is the rs1800629 SNP. This SNP, located at − 308 position with regard to the TNF promoter region, replaces guanine (G) with adenine (A), with the allelic types − 308 G/A, and has been linked to a variety of inflammatory condition and autoimmune diseases. The − 308 G/A SNP was investigated in AP and interconnected to the TNF level to figure out the responsibilities of TNF-alpha gene polymorphism in the pathogenesis of AP.

**Method:**

The current work design as a case–control study that involves 300 participant divided to 200 patients evaluated as (stable angina *n* = 100 and unstable angina *n* = 100) compared with 100 apparently healthy control subjects. The serum level of TNF-alpha was assessed via enzyme-linked immunosorbent assay (ELISA)/sandwich method. The genotype and allele frequency distribution of TNF-alpha rs1800629 gene polymorphism were investigated by TaqMan probe of allelic discrimination method.

**Results:**

The levels of TNF-alpha were significantly higher in patients with stable and unstable angina pectoris in comparison with controls. The deviation from Hardy–Weinberg equilibrium (HWE) of TNF-alpha genotypes was obvious in control and unstable angina pectoris groups. Moreover, the significant differences between patients with AP and controls under the five genetic models consider the association between TNF-alpha (rs1800629) − 308 G/A and AP with *OR* > 1. However, data analysis of allelic and genotypic of (rs1800629) − 308 G/A revealed higher significantly differences of GG homozygous and GA heterozygous proportions between stable angina patients and control. The A allele was more represented as etiological allele, and G allele was represented as protective allele. The serum levels of TNF-alpha were significantly higher in subjects with genetically mutated AA genotypes than in subjects with wild GG genotypes in the study groups. ROC curve analysis found the best cutoff value of TNF-alpha level was 77.25 pg/ml.

**Conclusion:**

As the results, our data observed a linked of TNF-alpha (rs1800629) − 308 G/A genetic variant with angina pectoris patients, and the A allele has been linked to the production or expression of TNF-alpha serum level and represented an etiological factor of angina pectoris.

## Background

The major cardiovascular phenotypes of ischemic heart disease (IHD) are coronary artery disease (CAD), myocardial infarction (MI), and stable/unstable angina pectoris. The etiology of IHD is largely acknowledged to be complicated and polygenic, with environmental factors such as high lipid levels, smoking, sedentary lifestyle, and infectious agents, as well as genetic susceptibility [1]. Angina is caused by the constriction of coronary arteries in individuals with atherosclerosis, resulting in insufficient delivery of blood and oxygen to actively respiring myocardial tissue [2]. This is caused by cholesterol plagues that block the blood vessels which deliver blood to the heart. Those who are suffering from angina pectoris are in danger of having a heart attack. The most prevalent symptom of angina pectoris is chest pain behind the breastbone. Angina can be categorized into two types: stable angina and unstable angina [3]. Cytokines, such as (TNF-alpha), promote signaling that is critical to the development and homeostasis of the immune system and plays an essential part in the cellular response to inflammation and injury. TNF-alpha induces protective mechanisms include host defense against infections and an inhibitory effect on carcinogenesis. Assuming TNF-alpha overload has negative modifications on tissues by creating a persistent inflammatory reaction and activated signaling pathways in the cardiovascular system, promote towards vascular dysfunction, atherogenesis, hypertensive, and unfavorable cardiac remodeling after myocardial injury [4]. The human TNF gene is a 7 kb DNA sequence consisting of TNF-A and TNF-B, which encode TNF-alpha and TNF-beta, respectively, and each includes 4 exons and 3 introns. TNF-alpha, a pleiotropic pro-inflammatory cytokine containing 233 amino acids, is produced by activated macrophages and encoded by the 4-exon of TNF gene at (6p21, 1585 bp) locus [5].

The presence of TNF-alpha polymorphisms has been linked to a variety of ailments, such as rheumatoid arthritis, types 1 and 2 diabetes, ankylosing spondylitis, sarcoidosis, and silicosis [6]. The documented relationship of the (rs1800629)- 308 G/A TNF-alpha variant with MI and CAD has thus sparked ongoing attention and given the potential impacts of the − 308 A allele on TNF-alpha level as well as the link between TNF-alpha and IHD with a logical hypothesis would be that − 308. A allelic variant is implicated in the development or increased risk of cardiovascular disease [7]. The purpose of this study in order to suggest that the (rs1800629) − 308 G/A TNF-alpha allelic type might be indicator to angina pectoris. It will also investigate whether the TNF-alpha gene polymorphism influences the serum concentration of TNF-alpha protein of Iraqi patients with AP documented by angiography.

## Methods

### Ethical permission

Ethical permissions were received from the subjects, and the study was also authorized by the Ethics Committees of the Biotechnology Research Center (BRC)/Al-Nahrain University according to the research ethics checklist of human subjects Ref. No. (M.B. 25). 

### Study subjects and selection criteria 

It was a case–control study; 300 participants including 200 subjects who had angiography that established the severity of (IHD), with criteria of 50% stenosis of at least one major coronary vessel due to atherosclerosis and assessed by two qualified cardiologists, and classify to 100 patients with stable angina and 100 patients with unstable angina were recruited from the “Iraqi center for heart disease at a surgical specialist hospital in Baghdad, Iraq” between February 2021 and August 2021 to participate in the present study. A prior history of IHD, MI, or AP is established by combining clinical data with a thorough study of medical records demonstrating diagnostic of electrocardiography (ECG) (significant elevation of ST segment), ejection fraction (EF%) as an assessment of the quantity of blood ejected by the left ventricle, and enzyme alterations. In addition, 100 apparently healthy subjects were those health issues and examination findings used to establish if the control participants who lived in the same regions as the cases were free of IHD and peripheral atherosclerotic artery disease.

### Sample size calculation

The G*Power software 3.1.9.7 was used to estimate the sample size of the current study. Test family (*t*-tests) and statistical test (means: difference between two independent means (two groups) were used for the analysis. The effect size *d* = 0.2, critical *t* = 0.73, *α* and *β* err prob = 0.2072386, and power (1-β err prob) = 0.81.

### TNF-alpha serum concentration identification

The serum level of TNF-alpha was assessed by (ELISA)/sandwich method using the kit’s manufacturer’s protocols (Abcam, USA, with a catalog number: ab181421) of one step assay with ranged from 31.25 pg/ml–2000 pg/ml and sensitivity 14 pg/ml.

### Genotype analysis of (rs1800629) − 308G/A SNP

This study has investigated the polymorphism (rs1800629) G/A substitution at position (− 308) in the promoter region of TNF-alpha gene. To extract genomic DNA from EDTA blood, the Wizard Genomic DNA Purification Kit was utilized (Promega, USA). The thermal profile of allelic discrimination approach employing real-time PCR was used to explore this polymorphism (Cephied, USA). The sequences of primers and probes were designed for the current study using the NCBI (National Center for Biotechnology Information) database and synthesized by Alpha DNA Ltd. (Canada).

**Table:** 

SNP ID	Primers/probes	Alleles	Region	Locus
(**rs1800629**)	F-5AGAAATGGAGGCAATAGGTTTTG-3 R-5ACTGATTTGTGTGTAGGACCCT-3 FAM-ATGGGGACGGG-MGB VIC-C ATGAGGACGGG-MGB	G/A	promotor	− 308

The reaction was conducted in a final quantity of 20 µl including 0.5 µl of each working SNP genotyping assay consist of primers (forward and reverse), probes (FAM and VIC), 10 µl TaqMan® Master Mix, 3 µl genomic DNA, and nuclease-free water and was used to complete the final volume of the reaction. Real-time PCR program cycling conditions have include initial denaturation at 94 °C for 10 min by 1 cycle accompanied by 5 cycles of denaturation at 95 °C for 20 s, annealing at 60 °C for 30 s, and extension at 72 °C for 40 s and then 35 cycles of denaturation 15 s, 94 °C/annealing 35 s, and 60 °C/extension 30 s, 72 °C.

### Statistical analyses

Analyses of data were performed using statistical comparison between angina pectoris and control subjects. TNF-alpha levels were provided as mean ± standard deviation (M ± SD) and were significant differences assessed using one-way analysis of variance (ANOVA). Observed and expected genotype was performed by chi-squared analyses used to test the deviation of genotype distribution from Hardy–Weinberg equilibrium (HWE) when *P* ≤ 0.05. Determined the differences of genotypes, allele frequencies, odd ratio (OR), and confidence interval (95% CI) between the groups via calculating the Fisher’s exact probability and to detect the attributable and preventive fraction in population by using statistical software (WINPEPI). The ROC curve was used to determine and predict the best cutoff value of TNF-alpha level.

## Results

### TNF-alpha serum level of the studied groups

The serum level of TNF-alpha was estimated among studied groups. The findings demonstrated a significant difference of the TNF-alpha concentration in stable and unstable angina and reached about 2-folds (86.33 ± 25.2, 95.83 ± 30.3) when compared to control group (35.98 ± 15.8) respectively. No significance has been indicated between patient groups. Table 1 displays all of the results.

**Table 1:** *P*-value of TNF-alpha (pg/ml) concentration among studied subjects

Subject groups	Concentration of TNF-alpha Mean ± SD	*p*-value vs. healthy subjects	*p*-value vs. stable angina	*p*-value vs. unstable angina
*Stable angina*	86.33 ± 25.2	–––-	–––-	0.144
*Unstable angina*	95.83 ± 30.3	–––-	–––-	–––
*Control*	35.98 ± 15.8	––-	0.002*	0.001*

^*^Significant value when *P* ≤ 0.05

### Characteristic and distribution of genotype and the allele frequency of TNF-alpha (rs1800629) − 308 G/A polymorphism

The genetic variation (rs1800629) -308 G/A of TNF-alpha was represented by three proportions (G/G wild or reference genotype, G/A heterozygous genotype, and A/A mutant genotype), all of which corresponded to two alleles (G and A). Based on categorization findings, there was a significant difference in patient with unstable angina and control between the observed and expected genotype frequencies (*P* ≤ 0.05) demonstrated by Hardy–Weinberg equilibrium (HWE) analyses; therefore, the accordance with the HWE was not established and deviated from the law. No significance was observed in patients with stable angina pectoris (*P* > 0.05). These findings were demonstrated in Table 2.

**Table 2:** Genotype and percentage frequency of TNF-alpha polymorphism (rs1800629)-308 G/A and their Hardy–Weinberg equilibrium (HWE) of the studied groups

Subjects group	Genotypes				HWE analysis of TNF-α genotypes	
		GG	GA	AA	*p*-value	*χ*^2^
Stable angina (*n* = 100)	Observed (*N* %)	28 (28%)	53 (53%)	19 (19%)	*P* > 0.05 (NS)	0.47
	Expected (*N* %)	29.70 (29.70%)	49.59 (49.59%)	20.70 (20.70%)		
Unstable angina (*n* = 100)	Observed (*N* %)	30 (30%)	33 (33%)	37 37(%)	*P* ≤ 0.01 (S)	11.34 Deviation
	Expected (*N* %)	21.62 (21.62%)	49.75 (49.75%)	28.62 (28.62%)		
Control (*n* = 100)	Observed (*N* %)	61 (61%)	28 (28%)	11 (11%)	*P* ≤ 0.05 (S)	6.41 Deviation
	Expected (*N* %)	56.25 (56.25%)	37.5 (37.5%)	6.25 (6.25%)		

*S*, significant; *NS*, non-significant. *χ*^2^, chi-square distribution

The results in Table 3 were illustrated in the genetic inheritance model of the genotype proportions of rs1800629 SNP. There were significant differences of the frequency distribution between patients with AP and controls in the (codominant, dominant, recessive, and over-dominant) inheritance model with *OR* > 1 and (*p* ≤ 0.05). Furthermore, there were significant differences in the A allele between patients with AP and controls (198 vs. 50) with (*OR* = 2.94; 95% *CI* 2.02–4.28).

**Table 3:** Genetic inheritance model genotypes and the association for allele frequency of TNF-alpha gene polymorphism (rs1800629) − 308G/Ain angina pectoris patients and control groups

Genetic model	Genotype of rs1800629 SNP	AP patients (*N* = 2 00)	Heathy control (*N* = 100)	OR: (CI: 95%)	*p*-value
Codominant	GG	58	61	Ref	-
	GA	86	28	1.94 (1.16–3.25)	0.012*
	AA	56	11	3.15 (1.57–6.31)	0.001*
Dominant	GG	58	61	Ref	
	GA/AA	142	39	3.83 (2.32–6.33)	0.000*
Recessive	GA/GG	144	89	Ref	
	AA	56	11	3.15 (1.57–6.31)	0.001*
Over dominant	GG/AA	114	72	Ref	
	GA	86	28	1.94 (1.16–3.25)	0.001*
Allele	G	202	150	Ref	
	A	198	50	2.94 (2.02–4.28)	0.000*

*P*-value ≤ 0.05, *OR*, odd ratio; *CI* 95% confidence interval; *significant difference; ref, reference

### TNF-alpha gene SNP (rs1800629 G/A) at -308 Locus

The estimation of the risk of angina pectoris suggested by the proportion level was shown in Tables 4 and 5. It was demonstrated that there have been significant differences between control and patients with stable angina in the state of homozygosity GG and heterozygosity GA genotype frequencies (*p* ≤ 0.05). In contrast, there was statistical insignificant in the state of homozygosity mutant AA proportion between the two groups (*p* > 0.05). With the prevalence of the G and A alleles, the significant results were noted across patients with stable angina and controls (*P* ≤ 0.05) (*OR* = 0.4; 95% *CI*: 0.26–0.61); (*OR* = 2.5; 95% *CI*: 1.64–3.82) in the G and A alleles, respectively.

**Table 4:** Genotype and allele frequencies of TNF-alpha gene polymorphism (rs1800629) -308G/A in a stable angina pectoris patients and control groups

Subjects’ groups	TNF-alpha gene SNP at position − 308 (SNP-ID: rs1800629)				
	Genotypes			Alleles	
	GG	GA	AA	G	A
Control (*n* = 100)					
No	61	28	11	150	50
%	61%	28%	11%		
Stable angina pectoris (*n* = 100 )					
No	28	53	19	109	91
%	28%	53%	19%		
OR	0.2	2.9	1.9	0.40	2.5
EF or PF	PF	AF	AF	PF	AF
95% CI	0.14–0.45	1.62–5.20	0.86–4.21	0.26–0.61	1.64–3.82
*P*-value	0.004*	0.001*	0.16	0.002*	0.002*

*OR*, odd ratio; *AF*, attributable fraction in population; *PF*, prevented fraction in population; *P*, Fisher’s exact probability

**Table 5:** Genotype and allele frequencies of TNF-alpha gene polymorphism (rs1800629) -308G/A in unstable angina pectoris patients and control groups

Subjects’ groups	TNF-alpha gene SNP at position − 308 (SNP-ID: rs1800629)				
	Genotypes			Alleles	
	GG	GA	AA	G	A
Control (*n* = 100)					
No	61	28	11	150	50
%	61%	28%	11%		
Unstable angina pectoris (*n* = 100)					
No	30	33	37	93	107
%	30%	33%	37%		
OR	0.27	1.7	4.7	0.29	3.45
EF or PF	PF	AF	AF	PF	AF
95% CI	0.15–0.49	0.69–2.31	2.26–9.99	0.19–0.44	2.26–5.27
*P*-value	0.001	0.53	0.002	0.000	0.000

*OR*, odd ratio; *AF*, attributable fraction in population; *PF*, prevented fraction in population; *P*, Fisher’s exact probability

Regarding the results of Table 5, there were significant differences in the GG and AA proportions across patients with unstable angina and control (*P* ≤ 0.05) (*OR* = 0.27; 95% *CI*: 0.15–0.49); (*OR* = 4.7; 95% *CI*: 2.269.99), respectively. The comparison was significant in the G and A alleles between the two groups of the (*OR* = 0.29; 95% *CI*: 0.19–0.44); (*OR* = 4.7; 95% *CI*: 2.265.27). Taking into consideration that the OR less than 1 meant the genotype or allele has a protective function in population and more than 1, the genotype or allele has an etiological or attributable function in a population.

### The serum level of TNF-alpha correlated with the (rs1800629) -308 G/A genotypes

**Fig. 1:**
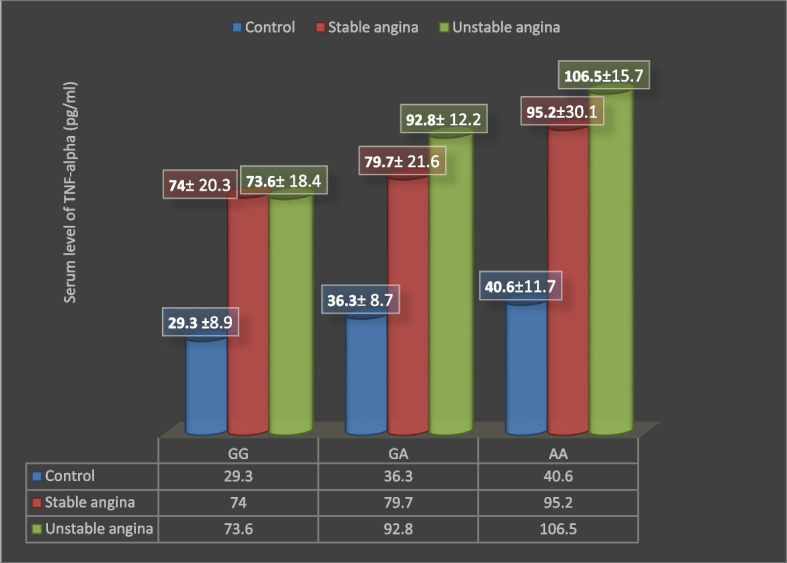
Serum level of TNF-alpha (pg/ml) pattern in control, stable angina, and unstable angina according to the (rs1800629)-308 G/A SNP proportions

The evidence of the associated TNF-alpha concentration and genotypes of the corresponding SNP was illustrated in Table 6 and Fig. 1, which include the three subject groups. This evidence indicated that the significant results appeared among the three groups in the same genotype (*p* ≤ 0.05) with the highest level reported in the patients of angina pectoris (stable and unstable), whereas the most significant was identified in the stable and unstable angina patients through the equivalent proportions with elevated level in the AA genotype.

**Table 6:** Serum level of TNF-alpha (pg/ml) distribution according to genotypes in the studied groups

Subject groups	Serum level of TNF-alpha (pg/ml) ) mean ± SD			*p*-value
	GG	GA	AA	
Control (*n* = 100)	29.3 ± 8.9A, a	36.3 ± 8.7A, a	40.6 ± 11.7A, a	0.42 (NS)
Stable angina (*n* = 100)	74 ± 20.3B, a	79.7 ± 21.6B, a	95.2 ± 30.1B, b	0.027 (S)
Unstable angina (*n* = 100)	73.6 ± 12.4B, a	92.8 ± 18.2C, b	106.5 ± 19.7B, b	0.033 (S)
*P*-value	0.03 (S)	0.000 (S)	0.04 (S)	

ANOVA test. Different capital letters mean there are significant differences between subject groups with the same genotype, and different small letters mean there are significant differences between genotypes with the same group. *NS* non-significant *P* > 0.05; *S* significant *P* ≤ 0.05

### ROC curve for prediction of TNF-alpha

**Fig. 2:**
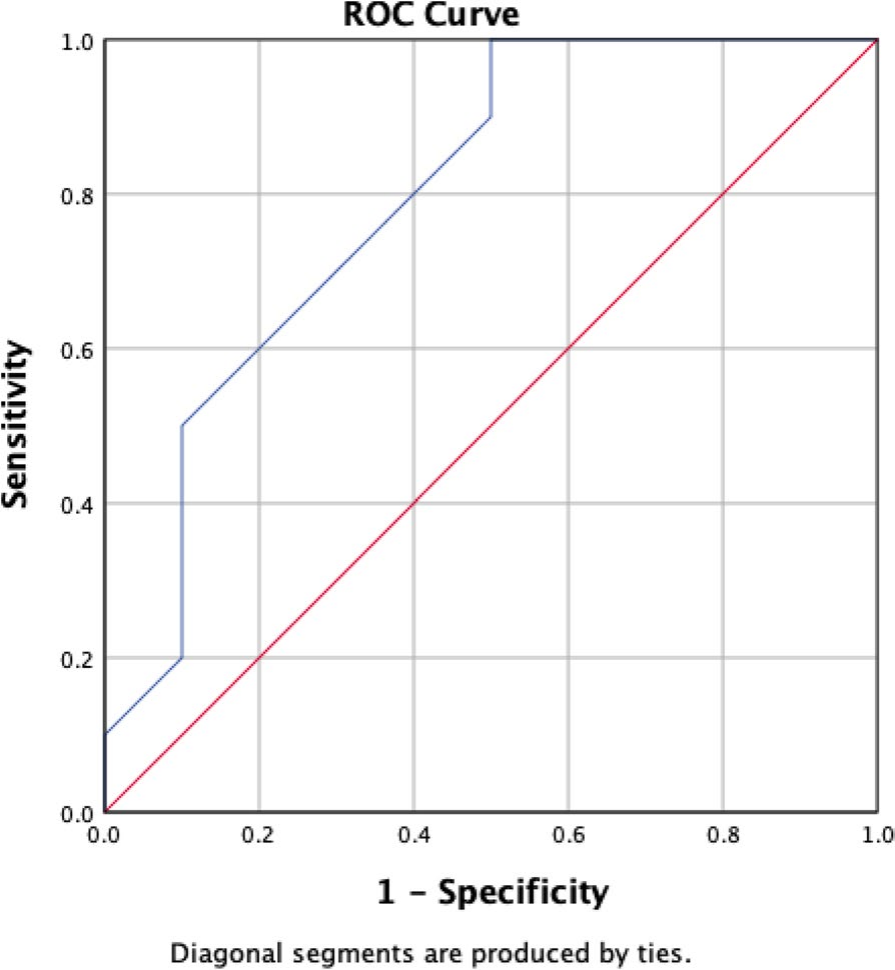
TNF-alpha ROC curve for predict angina pectoris vs. control

The ROC curve (Fig. 2) showed that the optimal TNF-alpha cutoff serum level was 77.25 pg/ml, where sensitivity was 70%, specificity was 30%, area under curve 0.795, and *CI*: 95% (0.594–0.996).

## Discussion

The objective of this study is to investigate the link between TNF-alpha (rs1800629) − 308 G/A genetic polymorphism and the risk or susceptibility to angina pectoris in its two forms in the Iraqi population, based on the supposition the IHD that include angina pectoris is a chronic inflammation condition. In this manner, the results suggest that the (rs1800629) − 308 A allelic variant was accompanied by changes TNF-alpha production and an increase in risk of angina pectoris in the Iraqi population. Several variations were identified in the promoter region of the human TNF gene, with the potential to establish structural changes inside regulatory regions, potentially disrupting the performance and management of TNF-alpha production or expression [8]. On the basis of the results of this study, it was revealed as the significant increase level of the TNF-alpha in the stable and unstable angina groups as compared with control. However, TNF-alpha is considered a pleiotropic cytokine that effects on the many activities of cellular system. Its effects are on the lipid metabolism, coagulation endothelial function, and insulin resistance [9].

Chronic inflammatory process is marked by elevated circulation levels of pro-inflammatory cytokines, adhesion molecules, and cytokine-responsive acute-phase proteins. Many of these inflammatory plasma indicators have been identified to predict future cardiovascular risk in patients with acute coronary syndrome. TNF-alpha levels in the blood have been linked to atherosclerosis of coronary artery risk factors such as dyslipidemia, overweight, and inflammation [10]. In a sample of stable and unstable coronary patients, TNF-alpha was significantly related to the severity of coronary disease. This connection could be an indication of chronic inflammatory load and a risk factor for severe coronary disease [11]. In this context, it should be noted that the (rs1800629) SNP has upregulate and elevate or modify the level of TNF-alpha protein. Therefore, the expression of cytokine was elevated by the existence of the − 308 A allele variant [9]. Consequently, [12] has clarified that the TNF-alpha transcription is increased by six- to eightfold when the single-nucleotide polymorphism − 308 G/A is present in the promoter region. Furthermore, polymorphisms that flank TNF genes have been shown to be meaningful biomarkers of TNF-alpha production and to influence transcription factor binding.

Further suggestion by [13] revealed that the TNF-alpha was a major contributor into types 1 and 2 diabetes predispositions in Iranian and Saudi patients, correspondingly. Furthermore, diabetes patients had three- to fourfold upregulate levels of circulating TNF-alpha than healthy controls. In a prior study on Egyptian patients, TNF-alpha was found to be strongly linked with cardiovascular risk factor such as TC, LDL-C, FBG, HbA1c, and creatinine [14]. According to [4], TNF-alpha enhances the inflammatory reaction and contributing to the clinical difficulties associated with cardiovascular disorder and autoimmune disease, both of which are closely linked to cardiovascular comorbidity. Hence, prior study has shown that the TNF-alpha is a significant cytokine involved in the advancement of different atherosclerotic conditions [15]. In this study, data analysis suggested five models of inheritance that differed significantly between all patients and control (*P* ≤ 0.05) with *OR* > 1. Thereby, the results sustained with [16] demonstrating that the individuals with the AA genotype or who held the A allele of the − 308 G/A polymorphism were more probably to have IHD. Taken together, these results indicate that TNF-alpha − 308 G/A polymorphism may have a significant impact on susceptibility to developing angina pectoris.

Genotypes and allele frequencies of TNF-alpha gene polymorphism showed a different genetic distribution between the studied groups. Both groups of patients with angina pectoris have significant differences of genotypes and G/A allele in comparison with control. These results can give information of the susceptibility for angina pectoris in the Iraqi patients by investigating the role of risk or protective alleles of (rs1800629) -308G/A at the promoter region of TNF-alpha gene. However, the possibility of attributable or etiological allele which has OR more than 1 exhibited risk factor in patients who is bearing the AA and/ or GA proportions and A allele. The results demonstrated that in the existence of the AA genotype, the concentration of TNF-alpha remained considerably higher in the examined groups, particularly in patients with unstable angina (106 ± 15.7). These observations imply that TNF-alpha may exhibit a role in the pathogenicity of angina pectoris among participants, which is genetically determined. Moreover, the (rs1800629) − 308 G/A has been the most studied variant of TNF-alpha, because numerous studies have found that the individual with the A allele possesses elevated rates of TNF-alpha in serum, significantly affecting the risk of developing CAD [17, 18]. Many case–control studies have been carried out to investigate the correlation between TNF-alpha -308 G/A polymorphism and cardiovascular disease, but the results have been equivocal. In 2007, a meta-analysis found no link between the G-308A polymorphism and CAD in groups primarily of European ancestry [19]. The study by [6] demonstrated that that there is no association between A allele of − 308 polymorphism of TNF-alpha gene and CAD in the Chinese population. Nevertheless, other meta-analysis published in 2011 found that the A allele of TNF-alpha gene imparted a 1.5-fold greater risk of developing CAD in Caucasians (AG + AA vs. GG, *OR* = 1.50; 95% *CI*: 1.23–1.77) [20]. To offer a more consistent and accurate evaluation of the link between the TNF-alpha − 308 G/A polymorphism and CAD risk, a meta-analysis of 36 datasets with 12,567 cases and 13,216 controls was performed. In this meta-analysis, high-quality research discovered a substantial link between the − 308 G/A polymorphism and CAD susceptibility in the general population [21]. TNF-alpha promoter genetic variations have been linked to TNF-alpha serum concentrations, which have been linked to first-time coronary heart disease and are a biomarker for repetitive cardiovascular events following a previous myocardial infarction [22]. Interestingly, the rs1800629 polymorphism, which might boost TNF gene transcriptional activity, associated with increased TNF-plasma levels in ischemic stroke etiology, and this result was confirmed by genetic association data analysis [23].

## Conclusion

To date, there were no data on the correlation of the TNF-alpha − 308 G/A with angina pectoris in the Iraqi population. This study provides a significant link between TNF-alpha -308G/A gene polymorphism and susceptibility to AP. Meanwhile, it could be suggested that A allele of − 308 G/A SNP of TNF-alpha is tightly correlated with a considerable risk of angina pectoris development according to the OR and the circulatory levels of TNF-alpha in the individuals with the mutant genotype. However, these findings of the critical concentration of TNF-alpha were recommend to be used as biomarker for angina pectoris.

## Data Availability

This article contains all of the data provided or processed during this study.

## Abbreviations

FAM: 6-Carboxyfluorescein
AP: Angina pectoris
MI: Myocardial infarction
TC: Total cholesterol
SNP: Single-nucleotide polymorphism
OR: Odd ratio
CI: Confidence interval
CAD: Coronary artery disease
TNF-alpha: Tumor necrosis factor alpha
ECG: Electrocardiographic
ELISA: Enzyme-linked immune sorbent assay
EF: Ejection fraction
HWE: Hardy-Weinberg equilibrium
NCBI: National Center for Biotechnology Information
